# Supplementary material for: Institutional capacity assessment in the lens of implementation research: Capacity of the local institutions in delivering WASH services at Cox’s Bazar district, Bangladesh
Source: PLoS One. 2024 Feb 13;19(2):e0297000. doi: 10.1371/journal.pone.0297000 (PMC10863886; doi:10.1371/journal.pone.0297000)
Supplement: Attachment — Submitted filename: Response to Reviewers.docx [file pone.0297000.s002.docx]

September 29, 2023

To: Editor, PLOS ONE

Re: Revision and responses to reviewer’s comments

Dear Editor,

Thank you for reviewing our manuscript entitled “Institutional Capacity Assessment in the lens of implementation research: Capacity of the local institutions in delivering WASH services at Cox's Bazar district, Bangladesh” for consideration for publication in the PLOS ONE.

We have made and highlighted revisions by using track changes mode to the manuscript based on editor’s and reviewer’s comments. Here are our point-by-point responses to the comments for kind consideration.

Journal Requirements:

When submitting your revision, we need you to address these additional requirements.

1. Please ensure that your manuscript meets PLOS ONE's style requirements, including those for file naming. The PLOS ONE style templates can be found at

<https://journals.plos.org/plosone/s/file?id=wjVg/PLOSOne_formatting_sample_main_body.pdf> and 
<https://journals.plos.org/plosone/s/file?id=ba62/PLOSOne_formatting_sample_title_authors_affiliations.pdf>

Response: Submission was done according to PLOS ONE’s style requirements.

2. We note that you have indicated that data from this study are available upon request. PLOS only allows data to be available upon request if there are legal or ethical restrictions on sharing data publicly. For more information on unacceptable data access restrictions, please see <http://journals.plos.org/plosone/s/data-availability#loc-unacceptable-data-access-restrictions>.

In your revised cover letter, please address the following prompts:

a) If there are ethical or legal restrictions on sharing a de-identified data set, please explain them in detail (e.g., data contain potentially sensitive information, data are owned by a third-party organization, etc.) and who has imposed them (e.g., an ethics committee). Please also provide contact information for a data access committee, ethics committee, or other institutional body to which data requests may be sent.

b) If there are no restrictions, please upload the minimal anonymized data set necessary to replicate your study findings as either Supporting Information files or to a stable, public repository and provide us with the relevant URLs, DOIs, or accession numbers. For a list of acceptable repositories, please see <http://journals.plos.org/plosone/s/data-availability#loc-recommended-repositories>.

We will update your Data Availability statement on your behalf to reflect the information you provide.

Response: Due to research ethics (data confidentiality), the data investigated during the current study are not made publicly accessible, however they are available from the corresponding author upon justifiable request. Additionally, ICDDR,B data policy supports the data availability upon request. Request for ICDDR,B research data should be addressed to Ms. Shiblee Sayeed, Senior Manager of Research Administration, at shiblee_s@icddrb.org.

3. We note that you have stated that you will provide repository information for your data at acceptance. Should your manuscript be accepted for publication, we will hold it until you provide the relevant accession numbers or DOIs necessary to access your data. If you wish to make changes to your Data Availability statement, please describe these changes in your cover letter and we will update your Data Availability statement to reflect the information you provide.

Response: Due to research ethics (data confidentiality), the data investigated during the current study are not made publicly accessible, however they are available from the corresponding author upon justifiable request. Additionally, ICDDR,B data policy supports the data availability upon request. Request for ICDDR,B research data should be addressed to Ms. Shiblee Sayeed, Senior Manager of Research Administration, at shiblee_s@icddrb.org.

4. Please include a separate caption for each figure in your manuscript.

Response: Thanks for the suggestion, edited in the manuscript.

5. Please ensure that you refer to Figures 1 to 2 in your text as, if accepted, production will need this reference to link the reader to the figure.

Response: Thanks for the suggestion, edited in the manuscript.

**Additional Editor Comments:**

Please revise your draft according to the reviewers’ (see attachment if any) and editor’s comments.
• I don’t really understand what you mean by “effective” in the title. Please explain it somewhere in the text. I can’t find the exact meaning of this.

Response: We have dropped effective from the title and revised it.

• I think the keywords are too much. Please reduce to a maximum of ~5

Response: Reduced to 5 key words

• Please correct some reference errors.

Response: Thanks for the suggestions, we have rechecked and confirmed the reference section.

• There is no space between words and citations in some locations, e.g., lines 478, 482, 484. Please correct the whole draft.

Response: Corrected across the whole draft.

• I think you don’t need a list of abbreviations.

Response: Omitted.

• For the declarations section, please see examples from other publications in PLOS One and adjust your draft accordingly.

Response: Declarations have been written in accordance with other PLOS One publications.

• I think the implications are still local. Please expand your implications to other areas as well.

Response: We have added a conclusion section where we have highlighted the implications:

“Institutional capacity assessment and sustainable improvement of the existing WASH program is an integrated approach for effective WASH service delivery. This paper generates evidence for the scope of required improvement that could help the entire WASH sector of Cox's Bazar district to gear them up to reach the SDG target of achieving access to adequate and equitable sanitation and hygiene for all by 2030. Key stakeholders recognised that if plans could be developed at the local level, then the implementation strategy would be more context-specific and appropriate. Average milestones were set to achieve the goal, although the respective Upazila team determined their milestone considering their WASH situation and local context. The research evidence suggests that decision-makers, donors, and development partners should consider learning from the WASH implementers and stakeholders about their existing capacity, gaps, and opportunities before planning for any WASH intervention in any particular area. This strategy could also be implemented in other low-and-middle-income countries to reach the SDG target by 2030.”

• You can add cite studies from other regions/countries about WASH institutions/stakeholders/governance to support your argument/compare your findings, e.g., <https://doi.org/10.3390/w13030314>

Response: Added a few that were found to be relevant.

• I don’t see the conclusion section. Please add it.
Response: Added.

Reviewers' comments:

Reviewer's Responses to Questions

**Comments to the Author**

1. Is the manuscript technically sound, and do the data support the conclusions?

The manuscript must describe a technically sound piece of scientific research with data that supports the conclusions. Experiments must have been conducted rigorously, with appropriate controls, replication, and sample sizes. The conclusions must be drawn appropriately based on the data presented.

Reviewer #1: No

Reviewer #2: Yes

2. Has the statistical analysis been performed appropriately and rigorously?

Reviewer #1: N/A

Reviewer #2: N/A

3. Have the authors made all data underlying the findings in their manuscript fully available?

The [PLOS Data policy](http://www.plosone.org/static/policies.action#sharing) requires authors to make all data underlying the findings described in their manuscript fully available without restriction, with rare exception (please refer to the Data Availability Statement in the manuscript PDF file). The data should be provided as part of the manuscript or its supporting information, or deposited to a public repository. For example, in addition to summary statistics, the data points behind means, medians and variance measures should be available. If there are restrictions on publicly sharing data—e.g. participant privacy or use of data from a third party—those must be specified.

Reviewer #1: No

Reviewer #2: Yes

4. Is the manuscript presented in an intelligible fashion and written in standard English?

PLOS ONE does not copyedit accepted manuscripts, so the language in submitted articles must be clear, correct, and unambiguous. Any typographical or grammatical errors should be corrected at revision, so please note any specific errors here.

Reviewer #1: No

Reviewer #2: Yes

5. Review Comments to the Author

Please use the space provided to explain your answers to the questions above. You may also include additional comments for the author, including concerns about dual publication, research ethics, or publication ethics. (Please upload your review as an attachment if it exceeds 20,000 characters)

Reviewer #1:

In the introduction there is reference made to the displaced persons and the potential impact on the resources, however the scope of the document is on the "host community"/Bangladesh population. In many areas the Forcibly Displaced Myanmar Nationals (FDMNs) represent the majority of the population. Specifically in the most water scare parts of the district. However, FDMN/refugees and the service delivery approaches to providing WASH services in the camps is ignored or excluded from the scope. This is ok, but it needs to be better defined 1) why Cox's was selected (vs other districts in BGD) and 2) why only focusing on host population is rigorous/insightful.

In the methods section there is reference to a similar assessment "Institutional mapping and analysis of WASH Services and Costs, WASHCost-CESS Working Paper No", however that approach also included a comparison between the institutional arrangements and service delivery models to quantitative data collected through case studies. It is unclear that without this type of additional, complementary data that the analysis carried out by the authors is very insightful at all. The authors mention in the last sentence of the manuscript that "In addition, assigned team collected the required information such as availability, accessibility and requisite for safe water, improved sanitation and hygiene services at their corresponding communities and institutions, prior to the day of workshop" However this information was not presented anywhere in the manuscript.

Response: Thank you so much for this comment. There was plenty of research conducted on the Rohingya refugee population inside the camp context and some of those papers already described the capacity of the local authority, development partners and local agency in terms of service delivery. Here are some examples:

Alam M-U, Unicomb L, Ahasan SMM, Amin N, Biswas D, Ferdous S, Afrin A, Sarker S, Rahman M. Barriers and Enabling Factors for Central and Household Level Water Treatment in a Refugee Setting: A Mixed-Method Study among Rohingyas in Cox’s Bazar, Bangladesh. Water. 2020; 12(11):3149. <https://doi.org/10.3390/w12113149>)

https://gbvguidelines.org/wp/wp-content/uploads/2019/04/Humanity-in-WASH-auditcapacity-Summary-4_3_19-to-CXB.pdf

https://www.pathfinder.org/wp-content/uploads/2022/04/PI-BD_MHM-Rohingya_2022-04-25.pdf

However, no research was done to explore the capacity of local government in delivering WASH services in the host community which was their primary responsibility prior to Rohingya influx. Therefore, we decided to collect data from the host community. In addition, we wanted to avoid duplication of data collection activity as there were multiple agencies who collected regular WASH data from the Rohingya camps.

Additional comments below and in the attached:

In the findings section there are instances when statistics are provided without reference. “DGHS participants mentioned that 90% of tubewells at the community clinics are not functional.” Specific sources should be provided in all cases where statistics are referenced.

Response: Thank you for this comment. However, this is a primary result from our capacity assessment workshop and mentioned by one of our respondents. Therefore, we cannot use a reference for results. In addition, we cannot verify a statistic provided by the respondent, nor use any reference.

I recommend that the document is restructured to move the information on the overall institutional arrangements to the introduction- or perhaps develop a background section. Much of the information presented in the “results” section is not specific to Cox’s Bazar and not linked to the data collected through the KIIs or workshops.

Response: Thank you for this comment. However, the overall institutional arrangements is our own interpretation which was generated from discussion with key stakeholders through KII and workshops. This interpretation may not only be specific to Cox’s Bazar and represent the situation of the entire country. Table 2 was generated from our own findings and this is closely linked with the previous paragraphs, so it will not be a good decision to rearrange this section. We hope that you will reconsider your suggestions.

solid waste is introduced on page 18 for the first time, but it is not included in the table 2. The table should be adjusted or the references to solid waste removed.

Response: Thank you so much for this comment. We have added waste in table 2 after revisiting our raw data.

“Recommendations from the participants” section is poorly organized. It isn’t clear who should be taking forward any specific recommended action, or if there is any general recommendation on changes to the institutional roles and responsibilities that might require a policy or legislative reforms.

Response: Thank you so much for this comment. Recommendations have been separated.

Reviewer #2: I take this opportunity to thank the authors for preparing this useful report. I have a few suggestions to share

Introduction : Page 5 line 76 the abbreviation DPHE needs to be put in full. In this section it would be useful to highlight more the WASH situation in that region from published literature. I suggest you also highlight how the private sector and other humanitarian organisations are involved WASH in this region seeing you mention them as lacking awareness on WASH policies.

You mention the DPHEs roles, do these involve formulation and awareness creation of the national policies, plans, and frameworks concerning water and sanitation? I think it would be useful background information for the reader to understand.

Response: Thank you so much for this comment. We have elaborated DPHE in the first instance.

We have included the situation of the Cox’s Bazar on pages 14-16 under “WASH conditions in the context of Cox's Bazar”. We have described a little bit about the current WASH scenario of Cox’s Bazar in the background also. If we want to add more on WASH from published literature, the length of the masnucript will be longer.

Methods
This section is clearly written. A few comments;
The authors reports that a convenient sampling method was used to identify institutions fully or partially involved in WASH-related activities in Cox's Bazar district. I propose a bit more information on how the partial or full involvement was assessed.

Response: Thank you so much for this comment. We have edited the sentence as follows:

“A convenient sampling method was used to identify potential participants from those institutions.”

I suggest the authors correct the referencing errors on page 8 line 152 and 154

Response: Referencing errors corrected.

Results
In this section is propose the authors set up this into two main sections;
One section to describe the setting/context and another to describe the key themes/ findings from the research activities. As it is its challenging to follow at what point the findings of the interviews begin to be described.
Table 2 a very useful table that describe the key actors. However, its is a rather long table, i suggest some edits to the table to make it easier for the reader to follow.

Response: Thank you so much for this comment. We have edited the results section and Table 2.

I suggest the authors correct the referencing errors on page 8 line 178 and 179
The main results and thematic areas are well described, i am curious to find out if there were any positive findings or reports by the participants from the interviews and workshops. As it stands the authors highlight only the challenges.

Response: Referencing errors corrected.

Discussion
This section is well written. I propose the authors, set up the discussion to highlight how their findings sit in light of other assessments that have been done in this area or similar settings. Are there things that are unique to this setting ?
I also propose the authors describe /suggest how these WASH policies can be better developed and utilised in such settings seeing there are different actors with different roles.

Response: Thank you so much for this comment. We have edited the discussion section as suggested and also added a conclusion section.

Include all the abbreviations used in the report in the list of abbreviations
A minor correction on the figures , both are labelled figure 1 and also these figures were not described/highlighted in the main text

Response: List of abbreviations have been suggested by the editor to be removed.
Figures have been corrected and referenced.

6. PLOS authors have the option to publish the peer review history of their article ([what does this mean?](https://journals.plos.org/plosone/s/editorial-and-peer-review-process#loc-peer-review-history)). If published, this will include your full peer review and any attached files.


If you choose “no”, your identity will remain anonymous but your review may still be made public.


**Do you want your identity to be public for this peer review?** For information about this choice, including consent withdrawal, please see our [Privacy Policy](https://www.plos.org/privacy-policy).

Reviewer #1: No

Reviewer #2: No
